# Emergency Department Closures and Patient Outcomes: Scoping Review of Impacts on Care, Equity, and System Performance

**DOI:** 10.5811/westjem.53973

**Published:** 2026-06-28

**Authors:** Zahra Ridha, Adam Steiner, Helena Son, Suneel Upadyhe, Stephenson Strobel

**Affiliations:** * McMaster University, Michael G. DeGroote School of Medicine, St. Catharines, Ontario, Canada; † McMaster University, Division of Emergency Medicine, Hamilton, Ontario, Canada; ‡ McMaster University, Department of Family Medicine and Economics and Health Policy, Hamilton, Ontario, Canada

## Abstract

**Introduction:**

Emergency department (ED) closures have become increasingly common across health systems worldwide, reflecting mounting pressures from staffing shortages, resource constraints, and rising patient demand. Closures, whether temporary or permanent, pose potential risks to timely access to emergency care and may significantly impact patient outcomes and healthcare system performance. Despite growing attention from policymakers and the public, academic evidence on real-world impacts of ED closures remains fragmented. In this scoping review we compiled and summarized the current literature directly addressing the impacts of ED closures.

**Methods:**

Using the Preferred Reporting Items for Systematic reviews and Meta-Analyses extension for Scoping Reviews criteria, we conducted a search of PubMed.gov, the Cumulative Index to Nursing and Allied Health Literature, and Web of Science databases for papers published in English after the year 2000 that directly addressed ED closures. We screened these papers by title and abstract. This search yielded 15 studies for which we then conducted a forward and backward citation search, ultimately producing 22 unique papers. Data from these papers were initially extracted into a spreadsheet and then organized into thematic tables based on the impacts they discussed.

**Results:**

Our search yielded 1,725 papers, and we ultimately included 22 of these (1.3%). Most studies were observational or quasi-experimental, covering 147 unique ED closures, with some analyzing all United States closures (thousands). We found that we could organize the papers into four themes determined by the impact of ED closures had on the following: 1) patient outcomes (eg, mortality, likelihood of acute myocardial infarction treatment) (n = 14); 2) equity-deserving populations (n = 6); 3) alternate EDs that remain open (n = 10); and 4) on healthcare networks as a whole, including emergency medical services (n = 5). Most of the papers were observational or quasi-experimental in design and drew data from a wide scale of closures, from single-site to national ED closures (> 500 sites). The largest proportion (40%) of studies were based in the U.S.

**Conclusion:**

The literature demonstrated mixed and context-dependent impacts of ED closures. The ED closures consistently adversely affected mortality rates of patients with time-sensitive conditions when travel times or distances exceeded certain thresholds. Closures also disproportionately affected equity-deserving populations. However, the negative impacts were often mitigated in the long term, and were less pronounced in well-resourced systems capable of adaptation. There are key gaps in the literature regarding impacts on conditions that are not time sensitive, on marginalized populations, and on broader system-level performance, which should guide future research.

## INTRODUCTION

Emergency department (ED) closures, both temporary and permanent, have become increasingly common across many healthcare systems. These closures arise from complex, intersecting pressures that include staffing shortages, constrained hospital capacity, resource limitations, and surging patient demand. 1–3 In 2023 alone, 868 temporary or permanent ED closures were reported across Ontario, Canada, with rural hospitals being disproportionately affected. 4 Estimates of ED closures in the United Kingdom (UK) suggest that there was a net reduction of 8% in the number of operating EDs in the 10-year period leading up to 2012. 5 Similarly, in the United States, the total number of hospital-based EDs decreased from 4,500 to 4,460 from 2005 to 2015, while ED visits per hospital-based ED grew uniformly by 28.6% across urban and rural areas. 6 These ED closures often occur in regions with high-baseline healthcare needs and limited alternative access points. 7 For emergency physicians, these closures are not abstract administrative events. They reshape patient flow, redistribute acuity across neighboring hospitals, and alter the geography of access to care.

Emergency departments serve as the health system’s front line for unscheduled, acute care, and they function as a critical safety net for patients requiring rapid evaluation, stabilization, and disposition.^8^ They play a particularly essential role for patients with time-sensitive conditions, defined in this review as clinical presentations in which delays in ED-based assessment, diagnostic testing, or definitive intervention are associated with increased morbidity or mortality. These include conditions requiring rapid triage and intervention (eg, trauma, stroke, sepsis, myocardial infarction), as well as urgent surgical or medical conditions where delays to disposition or intervention may meaningfully affect outcomes (eg, appendicitis, bowel obstruction). While the applicable time window for care varies by condition, ED closures may disrupt multiple stages of care delivery, including prehospital transport, triage, ED throughput, and access to definitive treatment, significantly impacting patient outcomes and broader health system performance.

Our aim in this review was to synthesize the existing literature on the impact of ED closures on health systems, patients, and the workforce, with a focus on outcomes that are directly influenced by disruptions in emergency care access and delivery. Specifically, we examine the following: direct patient health outcomes, including mortality, morbidity, and delays in time-sensitive care as measured by triage, treatment, disposition, or access to definitive intervention; impacts on equity-deserving populations, such as individuals experiencing homelessness and rural communities that disproportionately rely on EDs; spillover effects on nearby EDs and hospitals, including changes in patient volumes, acuity, and clinical resource strain; and system-wide consequences, including emergency medical services (EMS) transport times, interfacility transfers, and broader emergency care capacity.

By mapping the scope, methods, and findings of existing studies, we sought to clarify how ED closures affect emergency care delivery and to identify critical knowledge gaps to inform future research and health system planning.

## METHODS

### Scoping Review

This review summarizes research findings and identifies research gaps in the current literature on the impacts of ED closures, 9 following the Preferred Reporting Items for Systematic reviews and Meta-Analyses extension for Scoping Reviews checklist. 10 We did not conduct a critical appraisal of the sources, as the studies included highly variable methods, making comparison difficult.

### Eligibility Criteria

We applied the inclusion and exclusion criteria outlined in Table 1 to select papers. We included only those that focused on the effects of ED closures. We excluded a sizable body of literature regarding hospital closures because they were not specific to the effect of ED closures. All the studies took place within developed countries and were published after 2000. This cut-off was applied to capture contemporary evidence that is most relevant to current policy and practice contexts. We did not include the grey literature, as our focus was on peer-reviewed papers, allowing for a more methodologically robust synthesis of the evidence. Much of the grey literature includes news and policy articles—sources that do not provide clear methods and outcomes for thematic comparison.

### Search Strategy

We are aware of only one previous academic review of ED closures: Chambers et al reviewed the effects of how changing individuals’ distance to the nearest ED due to an ED closure impacted patient mortality risk. 11 The authors found inconsistent evidence that mortality is impacted when an ED is closed and patients must travel further for urgent or emergent care. 11 Their review informed concept scoping but our strategy was independently developed to extend to other aspects of healthcare beyond patient mortality.

We searched PubMed.gov, the Cumulative Index to Nursing and Allied Health Literature and Web of Science (WoS) databases to collect a list of primary research papers on our subject published January 1, 2000–May 31, 2025. These databases were selected as they provide comprehensive coverage of health services, allied health, and multidisciplinary research, while ensuring feasibility of screening. We used the search terms outlined in Figure S1 and subsequently combined them using the Boolean terms ‘OR’ and ‘AND.’ We also manually screened the references of all the included papers (backwards citation search) to capture additional studies that the databases may not have included. We then used WoS to search for all papers that cited this pool of papers (forward citation search) and screened those for relevant papers that did not appear in our initial search. For papers that were not available in the WoS database, their citations were compiled using Google Scholar. One reviewer conducted database searches; title/abstract and full-text screening were performed independently in duplicate, with third-party adjudication for disagreements. (See “Selection Procedure”.)

### Selection Procedure

Using the systematic review tool Covidence, two reviewers (ZR and HS) screened the titles and abstracts based on our inclusion/exclusion criteria. A third reviewer (AS) resolved conflicts and finalized the list of included papers. Review of the full text was then completed by two reviewers (ZR and AS), and the principal investigator (SS) confirmed the final selection of papers.

### Extraction and Analysis of Data

Three reviewers (ZR, HS and AS) extracted data from the final list of 22 papers and organized the information into a table similar to Table S1. The extraction categories were created to provide a summary of each paper, collecting data on study design, purpose, methods, and results. We completed a thematic analysis of the extracted data to identify common concepts in the study outcomes. Themes were developed inductively from the extraction tables, double-coded by two reviewers, and refined through consensus meetings with the principal investigator, consistent with scoping review guidance on collating, summarizing, and reporting results. 9 Each theme had a table listing associated papers and their key features, such as the method, population, intervention, and outcomes (Table 2–5). Three reviewers each received a set of papers to organize into the thematic tables independently. The principal investigator (SS) verified the tables.

## RESULTS

**Figure 1:**
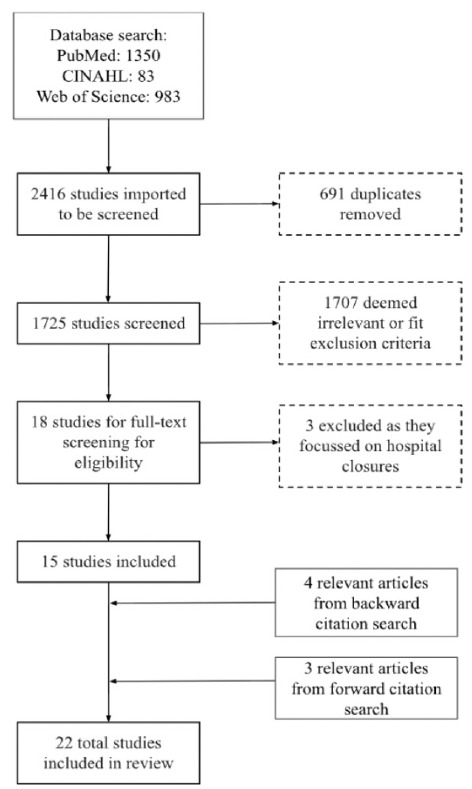
A PRISMA* diagrammatic analysis of the literature search conducted for a scoping review on the effects of emergeny department closures. Solid line boxes denote papers that were included; dotted line boxes represent excluded papers. *PRISMA, Preferred Reporting Items for Systematic Reviews and Meta-Analyses. *CINAHL*, Cumulative Index to Nursing and Allied Health Literature.

Our search yielded 2,416 results that we deduplicated (691 duplicates removed), producing 1,725 studies to be screened by title and/or abstract. We removed 1,707 that were irrelevant or fell under the exclusion criteria. We calculated the inter-reviewer agreement during title/abstract screening using the Cohen kappa, and it was deemed substantial (Cohen κ = 0.66). We completed a full-text review of 18 studies, subsequently excluding three papers. We completed a manual search of the references (backward citation search) of the remaining 15 studies and found four unique papers deemed relevant. We also completed a forward citation search, yielding six relevant papers. This process yielded a total of 22 unique studies that we included in this scoping review (Figure 1).

Our thematic analysis of the 22 included papers yielded four themes linked to ED closures: impacts on patient outcomes; equity-deserving populations; alternate hospitals that remained open; and broader healthcare networks (Tables 2–5). As we explored these themes, we purposely omitted a discussion on the background of each of the closures included (eg, the health system the ED was part of or demographics of the catchment area), unless they explicitly related to the theme, as a great deal of heterogeneity exists. This is mentioned as a limitation in our discussion.

### Theme 1: Impact of Emergency Department Closures on Patient Outcomes (14 papers; Table 2)

This theme generated the largest body of relevant literature and encompassed two subtopics: 1) access to care, including changes in distance and travel time; and 2) clinical outcomes, such as effects on mortality and time-sensitive conditions. Some papers examined the consequences of a single ED closure or a handful of closures (< 10), while others investigated closures across an entire region or country.

Many papers investigated how patient outcomes were affected by changes in distance/time to the next-closest ED after an ED closure. Early work by Shen and Hsia found that increases of less than 10 minutes were linked to modest rises in 30-day to one-year acute myocardial infarction (AMI) mortality that faded after three years. However, when travel times increased by > 30 minutes, elevated mortality persisted long term. 12 In a later study, these authors found that a minimum increase of 10 minutes in driving time raised mortality, both directly through treatment delays and indirectly through reduced access to care. 13 Avdic found similar results for AMI patients, although this effect disappeared a year after closure. 14 Hsia and Shen suggested that longer travel times (≥ 30 minutes) had stronger effects on mortality for patients with AMI, in a later study. 15

Not all studies, however, found a consistent link between increased travel time and higher mortality. Hsia et al reported no change in mortality associated with increasing distance to the ED for patients with time-sensitive conditions, although the majority of those patients experienced a travel increase of < one mile. 16 Likewise, Knowles et al found no evidence that ED closure was associated with an increase in mortality when the median increase in travel time to the nearest ED after closure/downgrading was nine minutes. 17 This suggests mortality rises only once travel delays exceed a threshold.

Other research focused on all emergency patients. Liu et al found that admissions to hospitals near an ED closure had 5% higher odds of inpatient mortality when compared to admissions to unaffected hospitals. 3 Lee et al explored temporary ED closures due to COVID-19 and found that these closures led to an increase in the in-hospital mortality rate of emergency patients (odds ratio of closure compared to non-closure group: 1.22 for nonEMS-use group and 1.23 for EMS-use group). 18

By contrast, other work did not identify clear harm for the general patient population. The centralisation of EDs in the UK, which resulted in multiple ED closures, was associated with improved outcomes. Price et al reported lower 60-day mortality, particularly among older adults, following the creation of a high-volume specialized ED in England. 19 In Denmark, Bogh et al found no association between ED closures and mortality, and Fløjstrup et al noted only a slight slowing of prior improvements in 30-day mortality trends after Danish ED reconfiguration. 20,21 Overall, there do not appear to be consistent results regarding mortality of the general patient population post-ED closure.

Beyond mortality, several studies assessed access to specific treatments. Shen and Hsia found that communities facing travel time increases > 30 minutes had higher rates of immediate primary percutaneous transluminal coronary angioplasty (PTCA). 12 Later work by the same authors showed that small increases in driving time, even those < 10 minutes, were linked to greater PTCA use once access to cardiac technology was controlled for. 13 These findings have been interpreted as reflecting differences in severity of presentation, with patients who arrive later being sicker and more likely to require urgent intervention. 12

Later studies found more complex patterns in treatment use. In 2019 Hsia and Shen examined the effects of ED closures on hospitals operating near capacity. They found that when a closure increased driving times by ≥ 30 minutes, the probability of receiving percutaneous coronary intervention (PCI) fell by just over two percentage points. 15 Conversely, the opening of a new ED that reduced driving times by ≥ 30 minutes was associated with higher PCI rates. 15 Just as with mortality, the effects of ED closures on treatment patterns appear inconsistent with some studies pointing to reduced access, while others suggest increased intervention due to delayed presentation.

### Theme 2: Effects of Emergency Department Closures on Equity-deserving Populations (6 papers; Table 3)

Within this theme, we classified papers into four subthemes: 1) effects on low-income regions; 2) sex differences in post-closure effects; 3) impacts on rural communities; and 4) impacts on individuals experiencing homelessness. Closures were found to disproportionately affect low-income and minority communities. Shen and Hsia found that low-income communities had a higher risk of a decline in ED access compared to high-income ones. 22,23 Areas that had a high proportion of Hispanic individuals also had a higher risk of decline in ED access. 22

Hansen et al highlighted sex differences in healthcare utilization after an ED decreased its hours and later closed. 24 They found a modest reduction in women’s use of healthcare services, while there were no significant changes for men. 24 There was a decline in women’s use of general practitioner services (telephone consults [*P* = .007], face-to-face visits [*P* = .020] and home visits [*P* = .009]) as well as a decrease in inpatient hospital utilization, after ED hours were reduced. 24

Rural communities also faced unique challenges after an ED closure. Shen and Hsia found that there was an average increase in driving time of 26 minutes for individuals from rural communities compared to 5 minutes for people who lived in urban communities. 22 Larsen et al. found that ED closures resulted in lower potential access to emergency care in rural communities in Ontario, Canada. These communities had more ED closures and less healthcare access overlap, making the impact of closures inequitably felt by these populations. 25

Gummerson et al studied the effects of closing an ED on homeless patients’ use of the nearby EDs that remained open. 26 They found that neighbouring EDs saw an increase in 15.46% of homeless patients vs 2.69% housed patients, demonstrating that homeless patients were disproportionately affected by this closure. 26 This evidence suggests that ED closures exacerbate existing inequities, with the most severe consequences borne by marginalized populations, including low-income or minority communities, women, residents of rural areas, and individuals experiencing homelessness.

### Theme 3: Impact of Emergency Department (ED) Closures on Alternate EDs that Remain Open (10 papers; Table 4)

Ten papers explored the effect of ED closures on nearby hospitals that remained open. We classified these papers into subthemes, including the following: 1) ED volumes and crowding; 2) staffing outcomes; 3) operational constraints; 4) financial impacts, and 5) healthcare utilization patterns. These studies tended to focus on the ability of these alternate ED sites to absorb patients who are diverted from a closed ED. They generally found short-term negative effects on nearby hospitals, followed by adaptations that limited these effects.

Emergency department closures led to a significant change in health outcomes at nearby hospitals that already had a high occupancy (increase of 2.39% in one-year mortality and 2.00% in 30-day readmission rates), but not at other bystander hospitals with lower occupancy. 15 The largest effects occurred in communities with limited resources, 15 such as crowded urban settings. These closures created adverse consequences across multiple dimensions of care. Patients who lost access to a closed ED and those seeking care at neighboring facilities were both affected, as closures at one site often led to crowding at others. 12 Closures may also have an impact on patients’ use of alternate EDs that remain open. Mustonen et al found that areas that experienced an increase in distance to ED services after closure saw a decrease in ED services use by 25% (*P* < .001). 27 However, Knowles et al and Bogh et al did not find similar spillover impacts in their studies of ED closures. 17,21

These studies also focused on how the effects of ED closures change over time. Negative effects may be limited, as EDs seem to be able to mitigate negative outcomes in the long term. Adaptation seems to be key to these results. Melnick and Fonkych and Melnick et al demonstrated how California hospitals have successfully adapted to the closures of EDs in recent years. 28,29 They showed that the supply of ED beds in California has grown by 17%, which far outpaced population growth. This is despite the number of hospital-based EDs falling between 1996–2007. 28,29 The counterbalancing force is that “remaining hospitals appear to have responded by adjusting their capacity to meet the growing demand for ED care.” 28

Shen and Hsia also found that the negative effects of ED closures on nearby hospitals tended to disappear after four years as healthcare resources were redistributed. 12 Avdic similarly found that negative effects at alternate hospitals are only statistically significant for approximately one year following a closure, and that eventually, “a well-functioning emergency medical service system may be able to offset the adverse quality effects [of a closure].” 14 The ED reconfigurations in Ireland were also found to cause short-term increases in crowding that generally resolved within a year. 30

Some papers observed the effects of ED closures on patient-use patterns of nonED health services. Mustonen et a. observed no increase in the use of local office-hour services, and a decrease in the total use of primary care physician services in areas that experienced an increase in distance to ED services after ED closure. 27 Knowles et al did not find consistent changes in urgent care attendance after ED closure. 17

### Theme 4: Impacts of Emergency Department Closures on Healthcare Networks (5 papers; Table 5)

Five of the studies included in this review looked at the impact of ED closures on larger healthcare networks. These were grouped into the following subthemes: 1) regional planning changes; 2) system-wide access issues; 3) policy responses; and 4) costs to regional governments and networks. In response to nearby ED closures in California, hospitals effectively adjusted their ED capacity so that there were no significant negative effects on emergency care throughout the region. 28 Additionally, network effects were studied after national-level reconfigurations of emergency medical care that involved closing EDs. In the UK, following the centralisation of emergency care and the closure of certain EDs, Price et al reported system-level benefits, including fewer readmissions (*P* < .01) and reduced ED reattendance (*P* <.01) that appeared by the second year after reconfiguration occurred. 19 Similarly, following a national reconfiguration of emergency care in Denmark that aimed to halve the number of EDs in the country, reductions in mortality occurred uniformly across the country, including regions where an ED closed and others where they stayed open. 21

Closures also have an impact on other emergency services. El Sayed et al found that closing a major ED in Boston, MA, led to a statistically significant increase in the mean ambulance turnaround time (0.89 minutes [*P* = .02]) despite overall ED volume being significantly decreased. 31 They noted that this increase was not clinically significant at the ED level, but that it may have been significant at the EMS-system level. 31 In South Korea, Park et al found that although EMS transport times (5.8 [SD 5.0] to 6.7 [5.5] minutes [*P* < .001]) were prolonged for certain populations near a closed ED in South Korea, there were no substantial delays or impacts seen throughout the entire EMS system. 32

## DISCUSSION

This review identified several patterns across heterogeneous study designs and health system contexts. First, patient outcomes may be correlated with ED closures and changes in distance or time to an alternative ED, particularly for time-sensitive conditions such as AMI but effects on the general ED population remain inconsistent. Second, closures impact care patterns in complex ways; for cardiac interventions, reduced access decreases intervention rates while delayed presentation paradoxically increases them due to greater acuity. Third, closures can disproportionately affect access to care for equity-deserving populations, including homeless, rural, and low-income populations, all of which already grapple with existing barriers to care. 33–35 Policymakers should be aware of these differential impacts and take measures to cushion the effects of closures for these at-risk populations.

Fourth, ED closures are associated with strain on alternate EDs. However, these effects are temporary, often lasting < 4 years as systems adapt through redistribution of resources. Effects vary by context, with well-resourced systems adapting better than already overstretched ones. Finally, national or regional reconfigurations of emergency care that involve ED closures do not appear to be associated with overall poor patient outcomes, and in some cases may even improve them. System-level restructuring can buffer against local harms, although effects on equity and frontline experience may still be significant.

Nevertheless, the interpretation of findings from this review is constrained by limitations that are common to the existing literature on ED closures. The only other previous academic review of ED closures that we identified in the literature was by Chambers et al, who noted several studies are at “relatively high risk of bias because of their observational design.” Indeed, most studies that we identified rely on observational designs using administrative data, with limited causal inference and high susceptibility to confounding variables such as staffing shortages, EMS reorganization, or hospital consolidation. Additionally, access to emergency medical care was most often measured as changes in travel time or distance, which may incompletely capture real-world barriers such as ambulance availability or patient decision-making.

Similar to our review, Chambers et al found inconsistent evidence that mortality is impacted when travel times change following an ED closure. 11 However, the main strength of our scoping review is its thematic analysis that extends our understanding of the effects of ED closures beyond mortality data to broader system-level effects, including impacts on nearby alternate EDs and entire healthcare systems. Additionally, it highlights gaps in the literature to guide future research, and it informs policy discussions in regions considering reductions in service.

These results have critical policy implications for proposed ED closures. Changes in travel time for patients upon closure of an ED are often shown to have a threshold effect on patient outcomes. Therefore, policymakers should consider defining maximum travel benchmarks when considering closures and ensure alternate EDs are resourced immediately. While transitions seem to eventually return patient mortality to a baseline level, there are associated increases in mortality especially in less well-resourced hospitals and EDs. Incorporating stakeholder input in these resource-allocation processes may also improve transparency and equity. 36

### Gaps in the Literature

This review highlights significant gaps in the current literature. First, most evidence on ED closures is observational, limiting causal inference. Although randomization of ED closures is not feasible, stronger application of quasi-experimental methods, such as difference-in-differences or regression discontinuity designs, would help establish more credible causal estimates of closure effects. Second, research is largely focused on mortality, with little insight into morbidity, patient experience, or outcomes for non-time-sensitive conditions that represent most ED visits. Including morbidity and other patient-centered outcomes would provide a more complete picture of closure impacts.

Third, evidence on vulnerable populations remains sparse. Evidence covers rural and homeless populations, but rarely LGBTQ, migrant, and racialized or indigenous groups that already face well-documented barriers to accessing care. 37–39 Fourth, economic impacts of closures are poorly understood. Within our included studies, we did not identify quantitative evaluations of local economic effects (eg, healthcare costs) following ED closures. This omission is striking given that closure decisions are frequently made with cost-saving goals in mind. Without rigorous evidence on local economic consequences, policymakers lack the information needed to fully weigh the trade-offs.

The final notable gap concerns the role of alternative models of care. Although policymakers often justify ED closures on the assumption that patients can be redirected to urgent care centers, walk-in clinics, or tele-emergency services, the existing literature provides little evidence on whether such substitution occurs. The few studies that do examine use patterns post-closure suggest that patients do not reliably shift into primary care or other local alternatives, raising the possibility that some care is simply foregone or delayed. Future research should investigate whether alternative care models absorb demand following ED closures, and under what conditions they can serve as effective substitutes.

## LIMITATIONS

This review has several limitations. Despite a comprehensive search of multiple databases, publication bias and missed studies remain possible. Excluding grey literature and non-English publications may limit generalizability. Also, the decision to exclude papers that broadly addressed hospital closures may have led to missed ED-specific data present in the articles. While our decision to include a broad set of EDs from different countries and healthcare systems (with articles detailing individual and nation-wide closures) was meant to provide a comprehensive summary of current research, this diversity also weakens the strength of our conclusions. Moreover, heterogeneity in methods and outcome reporting hindered cross-study comparison, causal inference, and a formal critical appraisal of the evidence base.

Additionally, we were unable to calculate an exact total number of unique ED closures represented across all studies. Our estimate is approximate, as several analyses drew from overlapping national datasets or reported aggregate closures without site-level detail. Our analysis was also primarily descriptive and thematic, without pooled quantitative analyses or meta-analytic techniques. This approach allowed us to capture a broad range of study contexts and outcomes but precluded statistical estimation of effect sizes or the identification of subtle associations that may emerge through quantitative synthesis. Finally, the dynamic and context-dependent nature of ED closures, which often occur alongside broader healthcare system changes, makes it difficult to isolate the direct impact of closures from other coexisting factors.

## CONCLUSION

In this scoping review, we aimed to synthesize the existing literature on the impacts of temporary and permanent ED closures on patient outcomes, equity, and health system performance. We found that ED closures have context-dependent effects. They are generally associated with worse outcomes for time-sensitive conditions when travel times exceed specifiable thresholds and disproportionately impact equity-deserving populations, including rural, low-income, and homeless communities. However, impacts on the general population and system-level mortality are mixed, with many health systems demonstrating adaptive abilities to mitigate harmful effects in the longer term, even when short-term harms can be common. Evidence on non-emergency conditions and economic outcomes remains limited. Next steps should focus on establishing evidence-based thresholds for acceptable travel distances post-closure and requiring mitigation and transition resources for affected communities. These frameworks are increasingly required at a time when the rate of ED closures is accelerating worldwide; otherwise, closure decisions will continue to be driven by fiscal pressures rather than population health needs.

## Supplementary Information





## Figures and Tables

**Table 1:** The inclusion and exclusion criteria used to screen for studies in a review on the effects of emergency department closures.

Inclusion criteria	Exclusion criteria
Primary research Published in English Studies in developed countries ED closures Temporary and permanent ED closure Focused on outcomes of closures	Reviews Study methodologies Hospital closures Urgent care closures Trauma center closures Ambulance diversions from EDs Studies published before 2000 News and perspectives articles Focus on ED crowding (not in the context of closures)

*ED*, emergency department.

**Table 2:** Review of papers included in theme 1: “Impact of emergency department closures on patient outcomes,” as part of a thematic analysis completed in a scoping review on the effects of emergency department closures.

Paper: Author, Year	Method	Population	Intervention	Control/comparison	Outcome	Primary finding
Hsia and Shen, 2019	Retrospective observational study	Patients with acute MI at bystander EDs in the U.S. from 2001–2013	ED closures and openings, as measured by increases in driving time	No change in driving time from bystander hospital and next nearest ED.	30-day, 90-day and one-year mortality rates. 30-day all-cause readmission rates. Treatment patients received (fibrinolytic therapy vs PCI).	Closures resulting in a driving time increase of ≥ 30 minutes led to increases in one-year mortality (2.39%) and 30-day readmission rates (2.00%). Inverse effects were found with ED openings with similar changes in driving time.
Liu et al, 2014	Retrospective observational study	Patients receiving care at hospitals near ED closures in California from 1999–2010	All ED closures in California 1999–2010	Patient admissions that were not impacted by an ED closure within a California Hospital Service Area	Inpatient mortality rates at hospitals near an ED closure.	Admissions to hospitals near an ED closure had 5% higher odds of inpatient mortality when compared to admissions to unaffected hospitals.
Park et al, 2024	Retrospective observational cohort study	OHCA patients in Ulsan, South Korea	One ED closure in Ulsan, South Korea	Adult nontraumatic OHCA patients pre-closure	Effect on survival outcomes of out-of-hospital cardiac arrest patients; effect on the EMS system	Closure of an ED did not significantly affect overall survival to hospital discharge.
Mustonen et al, 2017	Observational and quasi-experimental study (pre-post)	A Finnish municipality with a primary-care ED closure.	ED closure in Finland	Nearby area without an ED closure	Change in monthly visits to primary and secondary care EDs, office-hour clinics; monthly mortality	The use of ED services decreased in nearby areas by 25% following closure, with no corresponding increase in primary care usage or mortality.
Hsia et al, 2012	Nonconcurrent cohort study	Patients admitted to hospital for time-sensitive conditions in California (1999–2009)	Increased distance to nearest ED after closure	Patients with time-sensitive conditions experiencing no change in distance to the ED.	Inpatient mortality due to time-sensitive conditions.	No association between small increases in distances to the nearest ED and inpatient mortality from time-sensitive conditions.
Bogh et al, 2023	Pre-post population-based cohort	All Danish adults with unplanned ED contacts in 2008 and 2016	Centralization of EDs (reduction from 40 to 21 sites)	Municipalities with constant, lost, or never had EDs	Changes in ED travel distance, ED use, overall mortality, and AMI mortality	No association between ED closures resulting in greater travel distance and increased in-hospital mortality.
Avdic, 2016	Quasi-experimental observational study	Swedish residents < age 74 who experienced an AMI between 1990–2010	Increased distance to emergency care access after emergency hospital closure	Patients with no change in hospital distance – or a shorter travel distance post-closure – during the study period.	Post-AMI mortality Changes in survival based on proximity to emergency hospitals	The probability of surviving an AMI decreases by 2% for every 10-km increase in driving distance in the first year following a closure, but this effect fades after one year.
Shen and Hsia, 2016	Observational cohort study	1.35 million Medicare beneficiaries (U.S.) with acute MI from 2001–2011	Permanent ED closures, resulting in increased driving time to the next available ED	No change in driving time to the nearest ED	30-day, 90-day, 1-year mortality	Patients with a driving time increase of > 10 minutes to the nearest ED had a significant increase in mortality from AMI.
Shen and Hsia, 2012	Quasi-experimental retrospective cohort study	1.49 million Medicare beneficiaries hospitalized with acute MI in the U.S. between 1996 and 2005	Increased driving time to the nearest ED due to a local ED closure	No change in driving time (stable ED access throughout the study period)	Changes in mortality	Small increase in 30-day to 1-year mortality rates among patients experiencing a < 10-minute increase in driving time. Substantial increase in long-term mortality with a > 30-minute increase. Most of these effects are transitory and eventually disappear after four years.
Lee et al, 2021	Cross-sectional study	94,360 patients presenting to 46 EDs in South Korea between January–April 2020	Temporary closure of 6 major EDs due to COVID-19 exposure	Periods with all EDs open	Pre-hospital time, ICU admission, length of stay, in-hospital mortality	Temporary closures led to an increase in in-hospital mortality rates among emergency patients.
Knowles et al, 2019	Controlled interrupted time-series analysis	Residents in 5 UK urban catchment areas	Closure/downgrade of 5 EDs (2009–2011)	5 matched control areas with no ED changes	Ambulance incidents, ED attendances/admissions, and case fatality ratios	No evidence that reorganization of emergency care was associated with increased population mortality.
Flojstrup et al, 2023	Interrupted time-series analysis	Patients with unplanned emergency contacts in Denmark between 2007–2016.	National ED reconfiguration (stepped-wedge design)	Pre-reconfiguration period	In-hospital and 30-day mortality with subgroup analyses on selected diagnoses	Emergency care reconfiguration was not associated with an improvement in in-hospital mortality, and was associated with a slight slowing in previous improvements of 30-day mortality
Shen and Hsia, 2016	National observational study	1.35 million Medicare beneficiaries with AMI from 2001–2011	Permanent ED closures leading to increased driving time to nearest ED.	ZIP codes with no change in ED access.	Access to cardiac technology (CCU, cath lab, CABG), treatments, and mortality (30-day, 90-day, year)	ED closures have substantial consequences on patient outcomes, especially for time-sensitive conditions. Driving time increases of > 30 minutes led to a 90-day mortality increase of 6.58%.
Price et al., 2020	Retrospective service evaluation	52,439 unscheduled adult admissions in the UK from 2014–2017.	Centralization of 3 EDs into a single Emergency Care Hospital.	Pre-centralization baseline year vs. Year 1 and Year 2 post-centralization	60-day mortality, probability of discharge per day, readmission, ED reattendance, length of stay	Centralization of emergency care resulted in decreased 60-day mortality, faster discharges, and fewer readmissions.

*AMI*, acute myocardial infarction*; CABG*, coronary artery bypass grafting; *CCU*, cardiac care unit; *ED*, emergency department; *EMS*, *emergency medical services*; *OHCA*, out-of-hospital cardiac arrest; *PCI*, percutaneous coronary intervention; *UK*, United Kingdom.

**Table 3:** Review of papers included in theme 2: “Effects of emergency department closures on equity-deserving populations,” as part of a thematic analysis completed in a scoping review on the effects of emergency department closures.

Paper: Author, Year	Method	Population	Intervention	Control/comparison	Outcome	Primary finding
Hansen et al, 2011	Before-and-after study with comparative analysis	Residents of Morsø municipality (N = 21,000), 1997–2003	ED hours reduced and then full ED closure	Residents of neighboring municipalities in Viborg County	Change in use of ED and other services (GP visits, telephone consults, hospitalizations) by sex and SES	Modest reduction in women’s use of healthcare services following ED closure, with no significant changes among men.
Gummerson et al, 2022	Retrospective chart review	Homeless patients at two Rhode Island, USA, EDs near a closed hospital	ED closure	ED utilization pre-closure	Post-closure increase in ED visits by homeless patients; greater impact vs housed patients	Homeless patients were disproportionately affected by an ED closure, with an increase in homeless patients seeking care at alternate EDs.
Shen and Hsia, 2012	Quasi-experimental retrospective cohort study	1.49 million Medicare beneficiaries hospitalized with AMI in the USA between 1996–2005	Increased driving time to the nearest ED due to a local ED closure (< 10 minutes, 10–30 minutes, > 30 minutes increase)	No change in driving time (stable ED access throughout the study period)	Travel time changes and mortality rates for rural communities	Patients with the largest increase in travel time, and worse outcomes, were disproportionately from rural communities.
Larsen et al, 2023	GIS-based population analysis	Population of the province of Ontario, Canada.	Closure of 14 EDs in Ontario.	Baseline (all EDs open)	Population access within 30-, 45-, 60-min travel thresholds to nearest ED	Rural communities had relatively more ED closures and less healthcare access overlap.
Shen and Hsia, 2010	Retrospective observational study	Population of the continental U.S. 2001–2005	Changes in geographical access to EDs between 2001–2005 in the continental USA	Communities with no deterioration in access to the ED.	ED access among rural communities, SES, and ethnic background	ED closures disproportionately affected travel times for rural populations (average increase of 26 minutes) than urban populations (average increase of 5 minutes). Areas with a high proportion of Hispanic individuals also had a higher risk of a decline in ED access.
Shen and Hsia, 2016	Observational difference-in-differences study	1.35 million Medicare beneficiaries with AMI from 2001–2011	Permanent ED closures leading to increased driving time to nearest ED.	ZIP codes with no change in ED access	Access to cardiac technology, and mortality measures in low-income populations.	Increases in driving time (>. 30 minutes) to nearest ED resulted in statistically significant increases in 90-day and 1-year mortality.

*AMI*, acute myocardial infarction; *ED*, emergency department; *GIS*, geographic information system; *GP*, general practitioner; *SES*, socioeconomic status.

**Table 4:** Review of papers included in theme 3: “Impact of emergency department closures on alternate hospitals that are still open,” as part of a thematic analysis completed in a scoping review on the effects of emergency department closures.

Paper: Author, Year	Method	Population	Intervention	Control/comparison	Outcome	Primary finding
Hsia and Shen, 2019	Retrospective observational study	Patients with AMI at all EDs with nearby ED closure/opening in the U.S. from 2001–2013	ED closures and ED openings	No change in driving time from bystander hospital and next nearest ED.	30-day, 90-day and one-year mortality rates; 30-day all-cause readmission rates; treatment patients received (fibrinolytic therapy vs PCI) at high-occupancy vs low-occupancy hospitals	ED closures led to significant changes in health outcomes at nearby hospitals that already had high occupancy, many of which already had limited resources.
Avdic, 2016	Quasi-experimental observational study	Swedish residents under age 74 who experienced an acute MI between 1990–2010	Increased distance to emergency care access after emergency hospital closure	Patients with no change in hospital distance during the study period.	Mortality post-AMI, and changes in survival based on proximity to emergency hospitals	Negative effects occur at nearby hospitals for approximately one year following closure.
Shen and Hsia, 2016	Observational cohort study	1.35 million Medicare beneficiaries (USA) with AMI from 2001 to 2011	Permanent ED closures, resulting in increased driving time to the next available ED	No change in driving time to the nearest ED	30-day, 90-day, 1-year mortality	Patients with a driving time increase of more than 10 minutes to the nearest ED had a significant increase in mortality from AMI.
Shen and Hsia, 2012	Quasi-experimental retrospective cohort study	1.49 million Medicare beneficiaries hospitalized with acute MI in the U.S. between 1996 and 2005	Increased driving time to the nearest ED due to a local ED closure	No change in driving time (stable ED access throughout the study period)	Changes in 30-day, 90-day, 1-year mortality	The negative effects of ED closures on nearby hospitals tend to disappear after four years.
Melnick and Fonkych, 2009	Mixed-methods trend analysis	California acute-care hospitals with licensed EDs	Statewide ED closures and capacity trends (1996–2007)	Time-series comparison of hospital ED supply vs population and ED visits	ED bed counts; number of operational EDs; ED visit volume; population size; hospital admission rate from ED; geographic access (travel distance); patient severity risk	The supply of ED beds in California grew despite the number of hospital-based EDs decreasing.
Melnick et al, 2004	Retrospective economic and capacity trend analysis	All acute care hospitals in California with EDs, 1990–2001	Changes in ED capacity at hospital level over time	Time-based comparison within hospitals: 1990–1995 vs 1996–2001.	ED utilization trends; ED capacity; distance to nearest ED; financial outcomes	Remaining hospitals responded to nearby closures by adjusting their capacity to meet demand.
Knowles et al, 2019	Controlled interrupted time series analysis	Residents in 5 UK urban catchment areas	Closure/downgrade of 5 EDs (2009–2011)	5 matched control areas with no ED changes	Ambulance incidents; ED attendances/admissions; and case fatality ratios	No statistically significant spillover effects on alternate EDs following a closure.
Lynch et al, 2019	Interrupted time-series analysis	All patients recorded as waiting for inpatient beds in four Irish regions (2005–2015)	ED closures and reconfigurations	Pre- vs post-closure trends in trolley numbers (patients waiting for beds)	Regional ED trolley counts as an indicator of overcrowding/bed pressures	ED reconfigurations led to short-term increases in overcrowding that generally resolved within a year.
Mustonen et al, 2017	Observational quasi-experimental study	Finnish patients in a municipality with ED closure.	ED closure in Finland.	Nearby area without an ED closure.	Change in monthly visits to primary and secondary care EDs; office-hour clinics; monthly mortality	No increase in office-hour visits and a decrease in total usage of primary care physician services in areas experiencing an increase in distance to the nearest ED.
Bogh et al, 2023	Pre-post population-based cohort study	All Danish adults with unplanned ED contacts, 2008 vs 2016	Centralization of EDs (reduction from 40 to 21 sites)	Municipalities with constant, lost, or never-had EDs	Changes in ED travel distance; ED use; overall mortality; and AMI mortality	No statistically significant spillover effects on alternate EDs following a closure.

*AMI*, acute myocardial infarction; *ED*, emergency department; *PCI*, percutaneous coronary intervention.

**Table 5:** Review of papers included in theme 4: “Impacts of ED closures on healthcare networks”, as part of a thematic analysis completed in a scoping review on the effects of emergency department closures.

Paper: Author, Year	Method	Population	Intervention	Control/ comparison	Outcome	Primary finding
Park et al, 2024	Retrospective observational cohort study	OHCA patients in Ulsan, South Korea	One ED Closure in Ulsan, South Korea	Adult nontraumatic OHCA patients pre-closure	Effect on survival outcomes of out-of-hospital cardiac arrest patients; effect on the EMS system	EMS transport times were slightly prolonged near the site of the ED closure, but there were no substantial delays that impacted the entire EMS system.
Bogh et al, 2023	Pre-post population-based cohort study	All Danish adults with unplanned ED contacts, 2008 vs 2016	Centralization of EDs (reduction from 40 to 21 sites)	Municipalities with constant, lost, or never had EDs	Changes in ED travel distance; ED use; overall mortality; and AMI mortality	Reductions in mortality occurred uniformly throughout the country following centralization, including in regions that experienced an ED closure.
Melnick et a., 2004	Retrospective economic and capacity trend analysis	All acute care hospitals in California with EDs, 1990–2001	Changes in ED capacity at hospital level over time	Time-based comparison within hospitals: 1990–1995 vs 1996–2001.	ED utilization trends; ED capacity; distance to nearest ED; financial outcomes	Following ED closures, California hospitals readjusted capacity such that there were no significant reductions in access throughout the state.
El Sayed et al, 2012	Retrospective EMS database analysis	Patients visiting a Level I trauma center in Boston, MA (2006–2009)	Consolidation of two functional EDs into one (closure of one ED)	Pre-ED closure baseline	Ambulance turnaround time; patient volumes transported by EMS; waiting times for patients transported by EMS; waiting times for walk-in patients	The ED closure led to a statistically significant increase in EMS turnaround time despite overall ED volume decreasing significantly.
Price et al, 2020	Retrospective service evaluation	52,439 unscheduled adult admissions in the UK from 2014–2017.	Centralization of 3 EDs into a single emergency care hospital.	Pre-centralization baseline year vs. Year 1 and Year 2 post-centralization	60-day mortality, probability of discharge per day, readmission, ED reattendance, length of stay	System-level benefits, including fewer readmissions and reduced ED reattendance, appeared two years after centralization.

*ED*, emergency department; *EMS*, emergency medical services*; OHCA*, out-of-hospital cardiac arrest; *UK*, United Kingdom.
