# Supplementary material for: Emergency Department Closures and Patient Outcomes: Scoping Review of Impacts on Care, Equity, and System Performance
Source: West J Emerg Med. 2026 Jun 28;27(4):899–912. doi: 10.5811/westjem.53973 (PMC13436648; doi:10.5811/westjem.53973)
Supplement: Supplementary file 1 [file wjem-27-899-s001.docx]

Table S1: Copy of data extraction form used in a scoping review on the effects of emergency department closures.

| Field | Study 1 | Study 2 | Study 3 |
| --- | --- | --- | --- |
| Background (context) |  |  |  |
| Purpose |  |  |  |
| Hypothesis (if applicable) |  |  |  |
| Methods and Research Design |  |  |  |
| Main Findings |  |  |  |
| Additional Findings |  |  |  |
| Discussion Points |  |  |  |
| Key Conclusions/Takeaway |  |  |  |
