# Supplementary material for: Emergency Department Closures and Patient Outcomes: Scoping Review of Impacts on Care, Equity, and System Performance
Source: West J Emerg Med. 2026 Jun 28;27(4):899–912. doi: 10.5811/westjem.53973 (PMC13436648; doi:10.5811/westjem.53973)
Supplement: Supplementary file 2 [file wjem-27-899-s002.docx]

**Figure S1. Search strategy used in a scoping review on the effects of emergency department closures.**

("Emergency Department" OR "Emergency Room" OR "hospital closure*" OR "ED" OR "Urgent Care" OR "Emergency Medicine" OR "bystander" OR "nearby") AND ("Closure*" OR "Close*" OR "Shut down" OR "shutdown" OR "shut-down") AND ("Spill over*" OR "spill-over*" OR "spillover*" OR "patient volume" OR "acuity" OR "overload" OR "surge" OR "crowd*" OR "utilization" OR "mortality" OR "wait time*" OR "length of stay") AND ("Canada" OR "US" OR "U.S." OR "United States" OR "Mexico" OR "North America" OR "Europe" OR "Germany" OR "France" OR "UK" OR "U.K." OR "United Kingdom" OR "Netherlands" OR "Switzerland" OR "New Zealand" OR "Sweden" OR "Denmark" OR "Finland" OR "Norway" OR "East Asia" OR "Japan" OR "South Korea" OR "Singapore" OR "Hong Kong" OR "South America" OR "Argentina" OR "Brazil" OR "Chile" OR "Uruguay")
